# Leveraging Large Language Models and Agent-Based Systems for Scientific Data Analysis: Validation Study

**DOI:** 10.2196/68135

**Published:** 2025-02-13

**Authors:** Dale Peasley, Rayus Kuplicki, Sandip Sen, Martin Paulus

**Affiliations:** 1 Laureate Institute for Brain Research, 400 Civic Ctr, Tulsa, OK, United States, 1 (918) 774 6582; 2 Tandy School of Computer Science, University of Tulsa, Tulsa, OK, United States

**Keywords:** LLM, agent-based systems, scientific data analysis, data contextualization, AI-driven research tools, large language model, scientific data, analysis, contextualization, AI, artificial intelligence, research tool

## Abstract

**Background:**

Large language models have shown promise in transforming how complex scientific data are analyzed and communicated, yet their application to scientific domains remains challenged by issues of factual accuracy and domain-specific precision. The Laureate Institute for Brain Research–Tulsa University (LIBR-TU) Research Agent (LITURAt) leverages a sophisticated agent-based architecture to mitigate these limitations, using external data retrieval and analysis tools to ensure reliable, context-aware outputs that make scientific information accessible to both experts and nonexperts.

**Objective:**

The objective of this study was to develop and evaluate LITURAt to enable efficient analysis and contextualization of complex scientific datasets for diverse user expertise levels.

**Methods:**

An agent-based system based on large language models was designed to analyze and contextualize complex scientific datasets using a “plan-and-solve” framework. The system dynamically retrieves local data and relevant PubMed literature, performs statistical analyses, and generates comprehensive, context-aware summaries to answer user queries with high accuracy and consistency.

**Results:**

Our experiments demonstrated that LITURAt achieved an internal consistency rate of 94.8% and an external consistency rate of 91.9% across repeated and rephrased queries. Additionally, GPT-4 evaluations rated 80.3% (171/213) of the system’s answers as accurate and comprehensive, with 23.5% (50/213) receiving the highest rating of 5 for completeness and precision.

**Conclusions:**

These findings highlight the potential of LITURAt to significantly enhance the accessibility and accuracy of scientific data analysis, achieving high consistency and strong performance in complex query resolution. Despite existing limitations, such as model stability for highly variable queries, LITURAt demonstrates promise as a robust tool for democratizing data-driven insights across diverse scientific domains.

## Introduction

### Overview

The scientific process relies heavily on the community of scientists reliably and efficiently generating, collecting, organizing, and analyzing data based on reasonable hypotheses and quality datasets. This process is based on complex information that derives from every experiment and every observation. However, these raw data can be challenging to engage with, their valuable insights hidden in complexity and at times significant volume and multimodality of collected data. The information contained in these datasets cannot just be observed, it must be processed and analyzed. While trained analysts can access the underlying patterns given sufficient time, the wealth of knowledge contained within these data sets remains inaccessible to the average person. Thus, unlocking the full potential of scientific data requires analytical tools and techniques that can distill complex information into insights accessible to both experts and nonexperts alike.

Large language models (LLMs) provide one possible solution to harnessing the valuable information hidden in large experimental data sets. These powerful artificial intelligence (AI) systems have demonstrated the ability to explain information. For example, GPT models have demonstrated remarkable abilities in explaining complex scientific concepts by generating coherent, human-readable summaries from vast data sets, making information more accessible to a broader audience. LLMs can tailor their explanations to various levels of expertise, providing concise summaries for the layperson as well as in-depth analyses for the scientist [1-3]. However, a critical gap remains—how do we get the LLM to “know” the information we want it to report to the user?

We introduce the Laureate Institute for Brain Research–Tulsa University (LIBR-TU) Research Agent (LITURAt), a research assistant that provides easy access to and efficient analysis of data sets through a user-friendly interface. Leveraging LLMs, LITURAt enables users of any scientific background to explore hidden relationships within data sets. Recognizing that understanding relevant literature is time-consuming for scientists and challenging for nonexperts, our system searches PubMed for abstracts pertinent to the user’s queries, enhancing the meaningfulness of the results. Consequently, the agent can answer specific questions and provide broader context from related research. Designed for both experts and novices, LITURAt allows scientists to delve into detailed analyses while offering laypersons clear and concise explanations, making complex scientific concepts more accessible.

### Background

LLMs are AI models trained on massive amounts of text data. They have demonstrated remarkable abilities in various natural language processing tasks, including text generation, translation, summarization, and question-answering. However, their application in scientific domains presents challenges due to their potential for inaccuracies and the need for precise, domain-specific information. Even the most advanced LLMs are prone to recall information from their training data inaccurately. While LLMs demonstrate impressive capabilities in general language processing, their very training methodology presents a challenge for ensuring factual accuracy in specific domain scenarios. The vast amount of data used for training can lead to a phenomenon known as “catastrophic forgetting,” [4,5] where the model prioritizes broader language patterns over the nuances of individual data points. This inherent limitation means we need a different approach other than simply training an LLM on a data set and expecting it to provide accurate responses to specific queries within that domain.

To combat this challenge, a recall system can be used to provide information to the LLM to help anchor its output to a correct context. However, for complex tasks requiring a multistep information retrieval process, where the answer to each query informs the next, a more sophisticated architecture is necessary. These systems are referred to as agents [6]. The architecture behind these agents typically shares 2 characteristics. First, they operate in a loop in which they “act” multiple times before returning an answer to a user. Second, they can use existing tools. These tools are subsystems that perform defined operations like a calculator or a search function. Tool use can be facilitated by many different implementations but almost all of them involve the agent generating a string in a specific format that the system can then parse as a command. Once the tool has finished running, the result produced is returned to the LLM and then the loop repeats.

**Figure 1.**
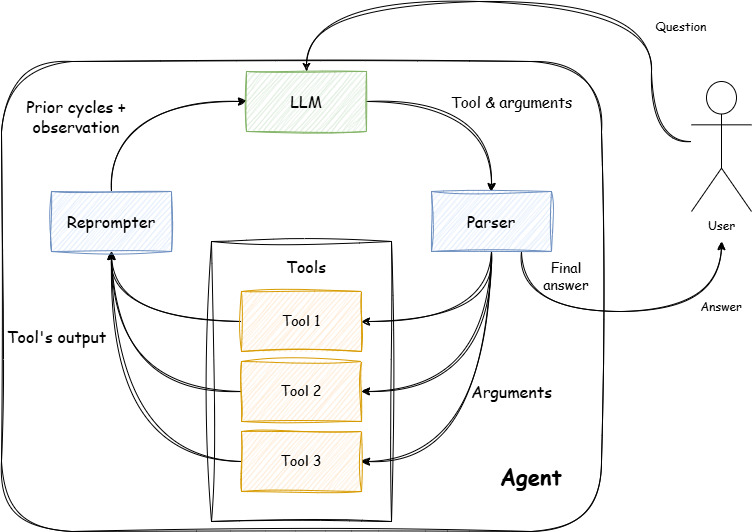
Generalized agent loop example. LLM: large language model.

Figure 1 shows an example of a tool-using agent’s core loop. When the user asks a question the LLM looks at the question and determines the first action that needs to be taken. The LLM generates a string that defines the next tool to be used and the arguments to be passed to it. The parser then reads the string and then calls the indicated tools with corresponding arguments. The selected tool runs with the specified arguments and returns some answers. The tool itself can be a piece of code, an application programming interface (API), or even another LLM-powered system [7]. The output of this tool is recorded, converted into a string, and added to the context window of the LLM as an observation by the reprompter. This reprompter is the mechanism responsible for the loop-like nature of the agent by running the LLM again with the newly added information. The loop repeats until the LLM decides it has enough to answer the question posed by the user. In that case, it generates a string that tells the parser to break out of the loop and present the user with the final answer.

To facilitate the development of this agent architecture, our system uses the LangChain [8] library which includes all the building blocks required for the creation of such agents. LangChain has been used in many similar applications [9-13] as it provides a framework in which to quickly construct agents.

Most current systems focus on providing interactivity with text files [14]. However, a large amount of scientific data are stored in the form of tables. The most common way that LLMs can interact with a local data set is through some form of search. Traditional searches have a low chance of success when applied to scientific data sets because the information requested often does not already exist and needs to be calculated. When this is the case a simple text-based search would return no useful information. Because of the calculations needed many common retrieval strategies fail and cannot provide the information the user requested to the LLM. Since the LLM is not given any context with which to answer the question there is no guarantee that the answer is anything more than a hallucination of the LLM.

## Methods

### Overview

**Figure 2.**
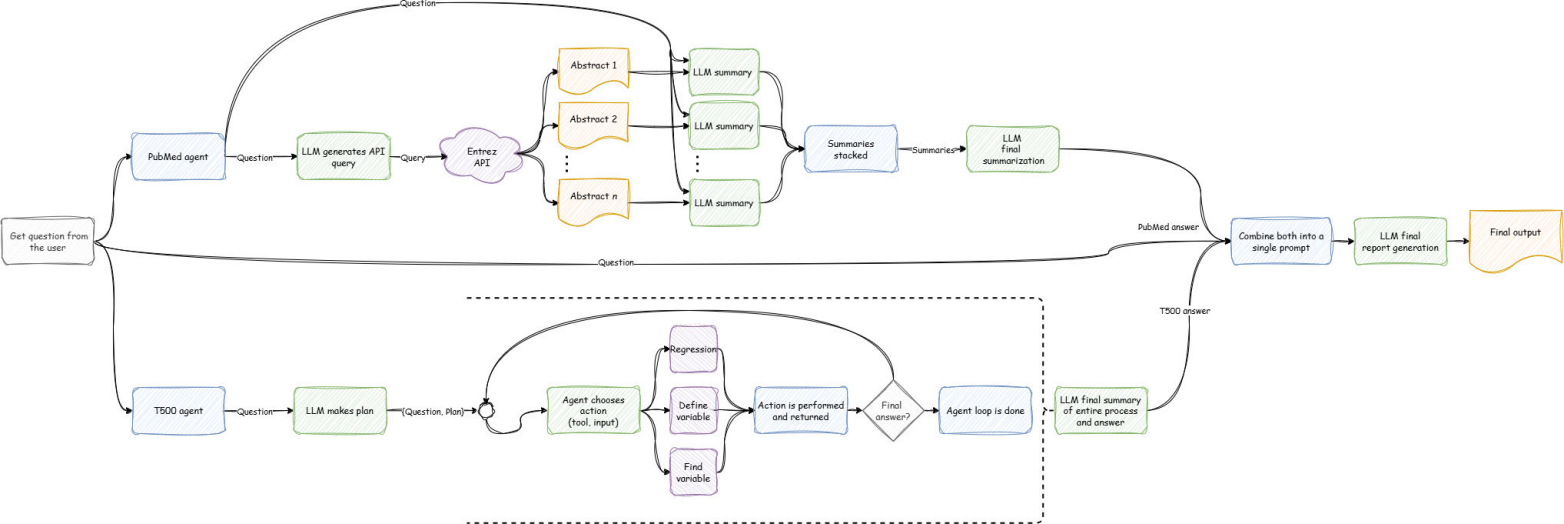
The architecture of LITURAt. API: application programming interface; LITURAt: Laureate Institute for Brain Research–Tulsa University Research Agent; LLM: large language model.

The goal of LITURAt is to answer questions about underlying relationships between the various entities in each data set and contextualize those answers using relevant literature. The user can pose questions such as “How strong is the connection between A and B?” or “Out of the set [A, B, and C] which is the best for predicting D?” Our system can then automatically perform the necessary analysis to answer this question while searching for relevant articles to provide the context to its answer. To accomplish this, the LITURAt uses a 2-pronged approach. One component looks at the local data while the other part summarizes the relevant retrieved literature. Figure 2 shows the overall architecture of LITURAt.

The first component is an LLM-powered agent-based system that uses local tabular data paired with a data dictionary to examine the relationships between underlying concepts. This allows the user to query about those concepts and the agent can answer questions about their relationships. The system uses an agent architecture like the one described above to allow for dynamic calculation of the requested information. The second component is an LLM-powered system designed to retrieve and summarize several published abstracts from PubMed. The summaries focus on information relevant to the user’s question’s context. The summarization happens along with the local retrieval system so that a user can see how local results fit into the current state of literature. Combining these systems gives the user the information extracted from the local data and informs them if this finding is consistent with other studies’ information. This can help to expose potential patterns that reinforce one’s own or others’ findings or it can help detect the presence of other factors that might cause the 2 study’s findings to differ.

For our implementation, we used Mixtral8 × 7b. While more powerful models exist, Mixtral8 × 7b has demonstrated the ability to be competitive with GPT-3.5 while being lightweight enough to run on midgrade commercial graphics processing units [15]. The combination of capability and lightweight performance makes Mixtral8 × 7b a practical choice for this task. While the initial goal was to develop a system that could answer questions of the format “What is the relationship between A and B,” due to the flexibility that the LLM-powered system provides, we found that it can answer much more complex questions. It has shown promising abilities to compare variables and relationships allowing it to tackle more complex questions about predictive abilities like the one mentioned above that contrasts 3 relationships to determine the best predictor. As the number of cycles through the core agent loop increases, we notice a decrease in the stability of the agent which impacts reliability. However, this only reaches a problematic level when asking a question that requires the agent to process 5 or 6 variables.

### Answering Questions From Local Dataset

#### Plan and Solve

Our agent uses a plan-and-solve approach. Its first step is to look at the question the user asked and the tools it has available. Before the agent begins its main loop, we have the LLM generate a plan to answer the question. The plan consists of which tools need to be used along with the order in which to use them. It also contains how the answer from one tool influences the arguments to other tools in future steps. This technique [16] helps guide the agent towards the solution while minimizing the chance of incorrect or unnecessary actions. It has been shown that adding a single planning step increases both the accuracy and efficiency of an agent by reducing the number of erroneous actions. This leads to agents taking a more direct path towards answering the question. We included the planning step to increase our system’s reliability when answering more complex questions.

#### Main Loop

The main body of the agent uses a loop following the design shown in Figure 1. The LLM is prompted to select tools using a JSON-style string [17] which is parsed by the system into tools and arguments. These 2 fields are then used to specify the tool the LLM chose from the set of available options and the arguments are then passed to this tool. When the tool finishes executing and returns a value, the system formats the output into another JSON-style object which is then turned into a string and passed back to the agent. This loop continues until the agent generates a JSON designating “Final Answer” as the tool then the system exits the loop, and the argument is the message returned as the answer to the question. As will be discussed later, this final answer is not used directly by our system as it provides too little information.

#### Abstracting the Dataset Behind Tools

One of the main advantages of having the model interact with the data via tools is the layer of abstraction the arrangement provides. The agent has no direct interaction with the data and does not need to be trained on any data so if the data set is changed, such as being updated, the agent’s ability to answer queries is unaffected. This also means that the system can be used on a variety of data sets just by modifying the tools that access the data set.

#### Tools

LITURAt uses 3 tools to answer the queries as follows.

##### Find Variable (Concept)

When this tool is passed a description of a concept as a query in the form of a string it searches the variable dictionary to find the variable in the data set that best represents the query.

##### Define Variable (Variable Name)

This tool simply retrieves the definition of the variable passed to it from the variable dictionary. This is used to give the agent more context leading to a more useful answer.

##### Linear Regression (Variable 1 and Variable 2)

Using the lm() function from the R language to create a linear regression model that uses variable 1, along with a set of manually-defined hardcoded controls, to predict variable 2. The summary() report contains the information to measure if there is a statistically significant relationship between the 2 variables.

### Find Variable Tool

**Figure 3.**
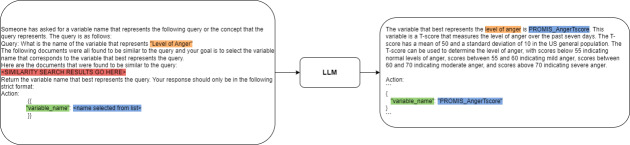
Variable search tool example. LLM: large language model.

One of the main factors that allows LITURAt to perform operations on data stored in tables is the ability to find variables that represent concepts given a variable dictionary which is another table that contains all the variables and their descriptions. The system needs to translate the description of the concept given by the user into the matching variable in the dataset. The system uses a similarity search metric over the definitions in the variable dictionary and sends the top 5 variables retrieved to the LLM model as candidates. The LLM uses the description provided to pick the one that best represents the concept. This step proved useful since there is no guarantee that the variable with the highest score on the similarity metric used in the search is the best choice to represent the concept. The LLM is used for its ability to semantically discriminate and select the best option. This extra layer of abstraction, instead of returning the search results directly to the main loop, reduces clutter and helps prevent confusion by only adding one variable name to the context of the main loop. When all the candidates are presented to the main loop, the chance of the LLM using the wrong one later increases. Figure 3 shows an example of the tool.

### Linear Regression to Measure the Strength of Relationship

In LITURAt*,* linear regression is used to measure the strength of a relationship between 2 variables. We used R language’s “lm” function to compare the 2 variables along with a standard set of covariates such as race/ethnicity, sex, employment, education level, income, and age. The result of the report was formatted with R’s “summary” function which provides a string representation that can be used by the LLM to interpret the results. The LLM model we used, Mixtral8 × 7b, demonstrated proficiency in interpreting these summaries and was able to extract the required information.

### Final Summary

When the loop returns a final answer*,* it frequently answers the initial query in the most basic form. Sometimes these answers can be as simple as “Yes, A is related to B.” This concise format does not contain enough information to create a meaningful report. Our solution to this sparsity problem is to take the agent’s entire loop history, the agent’s output as well as observations, and feed this into the LLM with instructions to generate a summary. The LLM is instructed to describe all steps the agent took, and all the knowledge gathered such as variable names and takeaways from the linear regressions. This can be seen in Figure 2 by the bracket surrounding the agent’s core loop. This final summary is an expanded version of the final answer, providing information such as the control variables used and the strength of the relationship between the concepts. We found that this gives a more comprehensive output leading to a better final report that allows the system to tailor the response to the user’s needs.

### Summarizing Relevant Literature

#### API Query Generation

To find papers relevant to the user’s question we had to search the abstracts available in PubMed. This search can be performed through the Entrez API [18]. The first step is passing the question to the LLM so it can generate a query formatted for the API. Passing this query through the API and extracting the top results gives us PubMed abstracts that pertain to the user’s question. The number of results retrieved can be adjusted and is a tradeoff between a more comprehensive search and the processing power required. In our experiments, we used the first 5 results returned by the API as relevant abstracts to the generated query.

#### Abstract Summary

To help distill the relevant information and prevent the size of the context window from becoming unmanageable, each abstract is passed through the LLM along with the initial question. The goal of this is to create a summary of the abstract within the context of the user’s query. We found that this helps to remove any extraneous elements of the abstract and compress the information that we want to return to the user. The result of this is a much shorter and denser string of text that contains the details in the abstract that directly relate to the question. Since these summaries are produced independently, they can be generated in parallel. Parallelization allows for the process to be scaled up without directly impacting the running time given a sufficiently powerful system.

#### Summary of Compiled Summaries

After all the abstracts have gone through the initial summarization process, the summarized versions are compiled together and passed through another summarization process much like in the first step. This time, the summary is over the entire sample of the literature we collected. Because we passed the user’s query to the LLM, the summary is focused on the relevant information. This 2-step summary process helps make the final summary useful when contrasting with the local results. It also helps filter extraneous information contained in papers that contain associated topics but do not provide a meaningful answer to the user’s question.

#### Focus on Lightweight Retrieval

One of the biggest motivations for the design of our retrieval system is to develop a lightweight method of adding context to the examination of local data. The system does not provide a comprehensive survey of all literature pertinent to the question. However, it provides enough information so that a user can see if the relationship seen in the local dataset was repeated in the literature or if there is a discrepancy between the local observations and past reports.

### Comparing and Final Report Generation

**Figure 4.**
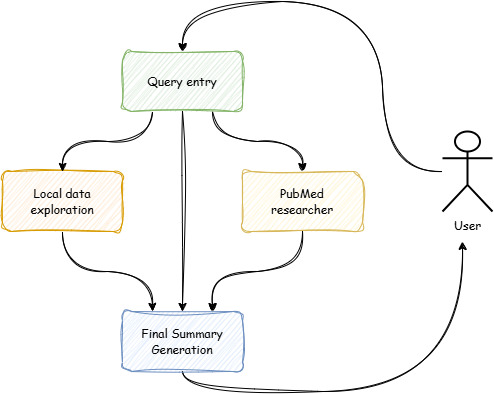
High-level architecture.

As seen in Figure 4, once both the local data exploration and the PubMed research are completed and their respective reports are generated, the final report is created. Both reports, along with the question, are passed to the LLM with a prompt to create a report stating the findings from both subprocesses and comparing them. The system generates a final report with a consistent predefined format as follows: (1) answer from local dataset, (2) answer from PubMed, (3) comparison of the 2 answers, (4) additional information, and (5) conclusion. An example can be seen in Multimedia Appendix 1.

This helps the model generate an accessible report in a consistent format. The goal is to convey the answers from both systems and discuss if the 2 support each other. This report is then given to the user as the answer to their question. If we wanted to use this system in the context of a chatbot, this report would be fed to the LLM inside the chatbot as context to aid in generating answers. The chatbot could then function as an interface to the human, providing an explanation at the desired level of detail. This process mirrors the way modern chatbots answer questions.

### Experiments

#### Overview

To test the effectiveness of our agent we used as our local dataset the Tulsa 500 (T-500), which is a subset of the Tulsa 1000 (T-1000) dataset [19]. The T-1000 was a naturalistic study that recruited and longitudinally followed 1000 participants, including healthy controls and treatment-seeking individuals with mood, anxiety, substance use, and eating disorders. Participants completed interviews as well as behavioral, biomarker, and neuroimaging assessments over the course of 1 year. The study aimed to determine how disorders of affect, substance use, and eating behavior were organized across various levels of analysis, including genes, molecules, cells, neural circuits, physiology, behavior, and self-report, to predict long-term prognosis, symptom severity, and treatment outcomes. The T-500 is a subset consisting of half the participants of the T-1000. We used this smaller selection as a testbed for the system.

#### Consistency

The first aspect we investigated was whether our agent reliably produced consistent results. It is critical to establish this since any further statistical analysis assumes that the given sample is representative. We measured 2 types of consistency that together quantify whether the system’s behavior is stable. First, we measured internal consistency, which we defined as the agent running with the same exact input multiple times and producing equivalent results each time. This measure captures the likelihood that the system will give different answers when asked the same question on separate occasions. The second type of consistency we compute is external consistency which measures the agent’s tendency to generate equivalent results when the phrasing of the question varies. We measure this to understand the risk of the agent changing its answer in response to minor changes in how the question was asked.

#### Internal Consistency

For the first type, internal consistency, we ran the agent 10 times on the exact same query. The predictors and outcomes were all pairs from a set of 4 variables. The reports generated by the system were then compared pairwise by Mixtral8 × 7b tasked with classifying the 2 reports as consistent or inconsistent. For this test, consistency was defined as containing the same key information with the only differences being in the format in which that information was presented. To evaluate consistency, we used Cohen κ coefficient for agreement to calculate the degree to which our agent agreed with itself when given identical input.

#### External Consistency

To evaluate the external consistency of our agent, we created a set of queries to which the agent should respond with the same information. For example, “What is the relationship between someone’s level of anger and their depression?” and “How does someone’s level of anger affect their depression?” should both produce the same key facts and thus both queries were considered requests for equivalent information. When answering equivalent questions, the system should mention the same basic facts about the direction and significance of the relationship. To determine the external consistency, we collected results from 5 requests for equivalent information across all combinations of 4 variables passed to the model. As with internal consistency, an LLM performed pairwise comparisons, with the result being used to compute the Cohen κ coefficient for agreement. The value of κ was interpreted as the level of agreement between the model and itself when the same question was phrased in different ways.

#### LLM-as-Judge

After evaluating the consistency of our agent’s output, we further evaluated the accuracy of the information produced by the agent. Any attempt to measure the accuracy of a literature review needs some reference with which to compare. Unfortunately, we could not find a proven baseline for literature review evaluation. Any system we created to help us with this test would be unproven and only lead to a comparison of 2 unknowns. Due to difficulties in establishing a ground truth for the literature review, our efforts focused on the local dataset exploration. To do this, we used GPT-4 as a judge. This practice has seen reliable results in multiple studies [20-22].

We tested the system with a batch of 192 questions, generated from 6 predictors and 7 outcomes, each phrased in multiple ways (Multimedia Appendix 2). All questions followed the basic format, “What is the relationship between A and B,” expressed in 1 of 5 variations to ensure homogeneity for easier analysis. For each question, we provided GPT-4 with ground truth data, including the query, relevant variables, and linear regression results. GPT-4 was then given LITURAt’s response from its local dataset module and asked to rate the accuracy of the agent’s answer on a 0-to-5 scale based on predefined guidelines shown in Textbox 1.

**Textbox 1. GPT-4 grading rubric.**

0 - no answer or completely unrelated

1 - attempt at an answer but completely wrong

2 - partially correct but missing key information

3 - mostly correct but with some errors

4 - correct but not comprehensive

5 - correct and comprehensive

## Results

### Consistency

Table 1 shows the results of our consistency experiments. Both tests gave results over 90% mark.

**Table 1.** Consistency results.

Experiment	Result
Internal	94.8%
External	91.9%

### Accuracy

Table 2 shows the result of the accuracy tests. The result from the accuracy evaluation demonstrates the potential of this approach. GPT-4 rated 80.3% (171/213) as entirely correct with most of the remainder being classified as mostly correct. Whether GPT-4 rated a response as “comprehensive” or not seems to depend on the level of detail and the requested level of detail was not specified or given to GPT-4 during the test.

**Table 2.** GPT-4 grading results.

Rating	Count	Frequency
1	4	0.018779
2	15	0.070423
3	23	0.107981
4	121	0.568075
5	50	0.234742

## Discussion

### Principal Findings

This investigation aimed to create an AI agent to aid the scientific process by integrating previous results in the literature with novel results from a new dataset. The results presented here highlight the potential of using AI-driven agents like LITURAt to bridge the gap between raw data and actionable insights in scientific research. By combining an LLM-powered system with a multistep tool-based approach, we were able to demonstrate both the flexibility and accuracy of this system in answering complex research questions derived from local datasets. Moreover, the consistency results—over 90% in both internal and external contexts—indicate that LITURAt can reliably produce stable outputs when faced with identical or similarly phrased questions. These findings suggest that, with further refinement, such systems could significantly enhance the efficiency of data interpretation, making scientific knowledge more accessible and usable across varying levels of expertise.

The findings from our study align with the growing body of research demonstrating the utility of AI-driven systems in scientific data analysis, particularly in enhancing accessibility and efficiency. Recent advances in LLMs have shown impressive capabilities in natural language processing tasks, from text generation to domain-specific question answering. However, much of the previous work has focused on static text sources, leaving a significant gap in their ability to handle complex, multimodal datasets like those common in scientific research. Studies have pointed out the limitations of LLMs in managing raw data, noting their propensity for inaccuracies or “hallucinations” when lacking sufficient domain-specific context. By incorporating agent-based architectures and dynamic data retrieval processes, LITURAt addresses this gap, providing more reliable and tailored responses by drawing directly from live datasets and relevant scientific literature.

Moreover, the introduction of a tool-based approach, which abstracts dataset interactions through specialized modules, represents an advancement over traditional search and retrieval methods commonly used in scientific applications. The capacity of LITURAt to use a “plan and solve” framework mirrors other innovations in AI research, such as the development of task-specific agents for complex problem-solving. This approach offers a more structured and interpretable framework, allowing LLMs to systematically approach multivariable questions in a way that traditional retrieval-based systems cannot. In doing so, our results contribute to a growing field that seeks to augment human-driven research with AI-powered tools capable of deep data analysis, reinforcing the idea that such hybrid systems will become crucial components of future scientific workflows. This integration of AI and human expertise holds significant promise for democratizing access to scientific knowledge, reducing barriers to data interpretation, and expanding the impact of research across disciplines. One of the most promising observations from this study was the way our system was able to scale up to more complex questions. The system could be asked for a simple relationship between A and B, but it could additionally answer a more complex related question such as determining if the relationship between A and B was stronger than the relationship between C and A. The ability to break larger tasks into smaller tasks and solve them step by step allows the system to tackle a wide variety of questions.

While the results of this study demonstrate the potential of LITURAt, several limitations must be acknowledged. First, the accuracy of the system heavily depends on the quality of the underlying dataset and variable dictionary, meaning that errors in data labeling or incomplete data could lead to misleading or incorrect results. Moreover, although the “plan and solve” approach improves performance in multivariable analyses, the system’s stability decreases when handling more complex queries involving 5 or more variables, limiting its scalability for larger datasets or more intricate analyses. Another limitation is the reliance on pretrained language models, which, despite their capabilities, are still prone to inaccuracies, particularly in domain-specific contexts where factual precision is critical. Although LITURAt uses external literature to validate findings, the summarization of PubMed abstracts is itself subject to the limitations of LLMs, including potential biases or oversimplifications in how scientific information is interpreted and conveyed.

In addition, the system’s performance was evaluated primarily using the T-500 dataset, a specific subset of the broader T-1000 dataset. As such, the generalizability of these findings to other types of datasets, particularly those with different structures or domains, remains uncertain. The reliance on Mixtral8 × 7b, a model chosen for its balance of computational efficiency and accuracy, also raises questions about whether more powerful models could yield better results, albeit at a higher computational cost. Last, while the consistency metrics are encouraging, the system’s ability to handle nuanced or ambiguous questions still requires further testing and refinement. These limitations suggest that while LITURAt holds significant promise, further optimization and broader testing across diverse datasets and contexts are necessary to fully realize its potential.

### Future Works

Several potential enhancements could improve system performance and reliability. First, integrating a rating mechanism for abstract summarization could prioritize more trustworthy sources based on criteria such as journal impact or author credentials. This would be particularly valuable in resolving disagreements between local results and published findings or inconsistencies across publications. In this study, linear regression was used to model relationships between variables. Future iterations could incorporate more sophisticated models to better capture complex relationships, enhancing analytical precision. Moreover, introducing an intermediate summarization step—where the LLM extracts only the most relevant information from statistical results—could reduce the buffer size in the main agent loop, improving stability for more complex tasks. The system architecture could also be scaled to incorporate multiple local datasets and literature repositories, providing more comprehensive and holistic results. Since the system’s modular design allows parallel execution of subsystems, this enhancement would require minimal architectural modifications and would not significantly impact runtime on capable systems. To further optimize performance, a mixed-model approach could be used: advanced models for critical computations and simpler models for basic tasks such as summarization. This strategy would enable better parallelization, reduce computational effort, and accelerate response times, enhancing overall user experience.

### Conclusion

In conclusion, this work has demonstrated the potential of a novel architecture that empowers both researchers and nonexperts to explore relationships within scientific datasets. LITURAt leverages the power of LLMs in combination with an agent-based system, allowing users of all backgrounds to interact with complex scientific data in a user-friendly manner. Our findings show that LITURAt not only exhibits strong consistency, ensuring reliable results, but also delivers accurate answers to a significant majority of user queries, further validating the viability of LLM-powered agents in extracting meaningful relationships from scientific datasets.

Moreover, the system shows promise in handling increasingly complex questions by automatically decomposing intricate queries into smaller, more manageable steps, broadening the scope of inquiries it can address. While challenges remain, particularly regarding model stability for more complex tasks and the limitations of the toolset, LITURAt represents a significant step forward in making complex scientific data accessible to a wider audience. These advancements pave the way for future systems that will further democratize access to scientific knowledge, enabling individuals from various fields and expertise levels to gain deeper insights from their data.

## Supplementary material

10.2196/68135 Multimedia Appendix 1 Example Laureate Institute for Brain Research–Tulsa University Research Agent output.

10.2196/68135 Multimedia Appendix 2 Templates and concepts used in tests.

## Abbreviations

AI: artificial intelligence
API: application programming interface
LIBR-TU: Laureate Institute for Brain Research–Tulsa University
LITURAt: Laureate Institute for Brain Research–Tulsa University Research Agent
LLM: large language model
T-1000: Tulsa 1000
T-500: Tulsa 500
