# Supplementary material for: Leveraging Large Language Models and Agent-Based Systems for Scientific Data Analysis: Validation Study
Source: JMIR Ment Health. 2025 Feb 13;12:e68135. doi: 10.2196/68135 (PMC11841814; doi:10.2196/68135)
Supplement: Multimedia Appendix 1 [file mental-v12-e68135-s001.docx]

Example LITURAt Output

1. Answer from T500

The T500 analysis found a statistically significant positive relationship between anger and depression, with anger explaining 45.26% of the variance in depression. Other factors such as Age, Education, Employment, Income, Race, and Gender were controlled for in the regression analysis and were found to be not significant.

2. Answer from PubMed

The PubMed analysis found evidence suggesting a relationship between anger and depression, with higher levels of anger associated with higher levels of depression.

3. Comparison of the two answers

The T500 analysis and the PubMed analysis both found a relationship between anger and depression, with higher levels of anger associated with higher levels of depression. However, the T500 analysis provided a more specific relationship, with anger explaining 45.26% of the variance in depression.

4. Additional information

The T500 analysis controlled for other factors such as Age, Education, Employment, Income, Race, and Gender, while the PubMed analysis did not specify if any other factors were controlled for.

5. Conclusion

The T500 analysis and the PubMed analysis both found a relationship between anger and depression, with higher levels of anger associated with higher levels of depression. However, the T500 analysis provided a more specific relationship, with anger explaining 45.26% of the variance in depression. The PubMed analysis did not specify if any other factors were controlled for, while the T500 analysis controlled for other factors such as Age, Education, Employment, Income, Race, and Gender.
