# Supplementary material for: Leveraging Large Language Models and Agent-Based Systems for Scientific Data Analysis: Validation Study
Source: JMIR Ment Health. 2025 Feb 13;12:e68135. doi: 10.2196/68135 (PMC11841814; doi:10.2196/68135)
Supplement: Multimedia Appendix 2 [file mental-v12-e68135-s002.docx]

Templates and concepts used in tests

Templates:

"What is the relationship between A and B?",

"How does A relate to B?",

"What is the connection between A and B?",

"How does A affect B?",

"Does A influence B?"

Concepts used as predictors:

"someone's level of anger",

"sleep disruption",

"BMI",

"someone's favorite drug being Methamphetamines",

"someone's sadness",

"Anxiety",

"depression"

Concepts used as outcomes:

"someone's level of anger",

"sleep disruption",

"BMI",

"someone's sadness",

"anxiety",

"depression"
